# Intrainsular connectivity and somatosensory responsiveness in young children with ASD

**DOI:** 10.1186/s13229-017-0143-y

**Published:** 2017-06-13

**Authors:** Michelle D. Failla, Brittany R. Peters, Haleh Karbasforoushan, Jennifer H. Foss-Feig, Kimberly B. Schauder, Brynna H. Heflin, Carissa J. Cascio

**Affiliations:** 1 Department of Psychiatry, Vanderbilt University Medical Center, Nashville, TN 37212 USA; 2 South Carolina Department of Mental Health, Columbia, SC 29202 USA; 3 Interdepartmental Neuroscience (NUIN) PhD Program, Northwestern University, Evanston, IL 60208 USA; 4 Seaver Autism Center for Research and Treatment, Icahn School of Medicine at Mt. Sinai, New York, NY 10029 USA; 5 Department of Psychiatry, Icahn School of Medicine at Mt. Sinai, New York, NY 10029 USA; 6 Department of Psychology, University of Rochester, Rochester, NY 14611 USA; 7 Vanderbilt Kennedy Center, Nashville, TN 37203 USA

## Abstract

**Background:**

The human somatosensory system comprises dissociable paths for discriminative and affective touch, reflected in separate peripheral afferent populations and distinct cortical targets. Differences in behavioral and neural responses to affective touch may have an important developmental role in early social experiences, which are relevant for autism spectrum disorder (ASD).

**Methods:**

Using probabilistic tractography, we compared the structural integrity of white matter pathways for discriminative and affective touch in young children with ASD and their typically developing (TD) peers. We examined two tracts: (1) a tract linking the thalamus with the primary somatosensory cortex, which carries discriminative tactile information, and (2) a tract linking the posterior insula—the cortical projection target of unmyelinated tactile afferents mediating affective touch—with the anterior insula, which integrates sensory and visceral inputs to interpret emotional salience of sensory stimuli. We investigated associations between tract integrity and performance on a standardized observational assessment measuring tactile discrimination and affective responses to touch.

**Results:**

Both the thalamocortical and intrainsular tracts showed reduced integrity (higher mean diffusivity) in the ASD group compared to those in the TD group. Consistent with the previous findings, the ASD group exhibited impaired tactile discriminative ability, more tactile defensiveness, and more sensory seeking (e.g., enthusiastic play or repetitive engagement with a specific tactile stimulus). There was a significant relation between intrainsular tract integrity and tactile seeking. The direction of this relation differed between groups: higher intrainsular mean diffusivity (MD) (reflecting decreased tract integrity) was associated with increased tactile seeking in the TD group but with decreased tactile seeking in the ASD group. In the TD group, decreased tactile defensiveness was also associated with higher intrainsular MD, but there was no relation in the ASD group. Discriminative touch was not significantly associated with integrity of either tract in either group.

**Conclusions:**

These results support previous findings suggesting a central role for the insula in affective response to touch. While both discriminative and affective touch and both somatosensory tracts are affected in ASD, the restriction of brain–behavior associations to the intrainsular tract and tactile seeking suggests more complex and perhaps higher-order influence on differences in tactile defensiveness and discrimination.

## Background

Autism spectrum disorder (ASD) is characterized by developmental impairments in social communication and restricted and repetitive behaviors [1], which often include differences in sensory responsiveness to environmental stimuli1 [2, 3]. Children with ASD may exhibit patterns of altered sensory reactivity such as hypo-responsiveness [4–6], hyper-responsiveness [7, 8], and/or sensory “seeking”—unusual interest in or fascination with specific sensory stimuli [9]. While these patterns manifest with considerable inter-individual variability and are likely to have different underlying genetic [10] and neural [11, 12] mechanisms, recent work has begun to describe how these patterns of behavioral reactivity to sensory stimuli co-occur within individuals [13].

Efforts toward untangling the neural basis of sensory reactivity patterns have focused on auditory and visual modalities, possibly because of their primacy to verbal and nonverbal communication skills affected by ASD. Far less work has been done to understand more proximal sensory systems such as touch, proprioception, and interoception. However, these systems are also of critical importance during social interactions, providing important cues about emotion, attachment, compliance, and intimacy [14]. Tactile responsiveness patterns specifically are linked to social deficits in ASD [6] and may have a central role in our earliest experiences of social communication. Specifically, infant–caregiver interactions that lay the foundation for social reward and secure attachment heavily involve the sense of touch [15, 16], which is at an advanced stage of development relative to other sensory systems in neonates [17]. It is during this early window in the first year of life that neural differences in ASD begin to emerge [18]. Thus, the developmental primacy of touch for behaviors relevant to ASD warrants further investigation.

While sensory reactivity is typically assessed with clinical observational measures, discriminative touch has also been increasingly measured in ASD using rigorous psychophysical methods that often give insight into neurobiological mechanisms. While there is not a clear consensus for impaired or enhanced discriminatory ability, some themes have emerged from this work (for a review, see Mikkelsen et al. [19]). Impaired performance on vibrotactile static detection and discrimination tasks suggests impaired lateral inhibition in the somatosensory cortex [20, 21], as do impairments in amplitude discrimination and the absence of expected effects of a habituating stimulus [20, 22]. These sensory indications of impaired cortical inhibitory mechanisms have been tied directly to reduced GABA in the sensorimotor cortex in ASD using spectroscopy [23] and support the excitatory/inhibitory imbalance hypothesis of ASD [24, 25]. The well-established autism candidate gene GABRB3 is associated with differences in tactile sensitivity [26, 27], providing further support for a role of GABA dysfunction in the somatosensory differences common in individuals with ASD.

Distinct but overlapping neural pathways support the discriminative and affective aspects of touch. Extensive work suggests that discriminative touch (e.g., touch used to determine the shape or texture of an object) is primarily mediated by the thalamocortical projections to the somatosensory cortex [28–30]. However, a separate system that is believed to mediate affective touch (e.g., touch used to elicit an emotional response or to communicate social affiliation) has recently been described. In this system, small-diameter, unmyelinated peripheral fibers, known as C-tactile (CT) afferents, respond preferentially to slow, stroking touch with light to moderate pressure, suggesting they are “tuned” to social/affective (in contrast to discriminative) touch [31–33]. Functional magnetic resonance imaging (fMRI) studies in patients lacking large-diameter myelinated tactile afferents demonstrate that CT fibers project selectively to posterior insular cortex [32, 34], a cortical target of multimodal sensory input that is associated with changes in affective state, including visceral sensation, temperature, and pain, further distinguishing this affective touch system from discriminative touch.

The insula is part of a complex cortical structure with a heterogeneous functional anatomy along its anterior-posterior axis. While the posterior insula receives somatosensory and visceral input, the anterior insula comprises heavily reciprocal projections with prefrontal and limbic regions [35, 36]. This functional organization may be the basis of a caudo-rostral hierarchical processing stream by which sensory cues are received and integrated with emotional signals to form progressively higher-order representations of proximal (tactile and interoceptive) sensory information and its affective significance [37]. This model is supported by fMRI evidence of strong connectivity between the anterior and posterior insula [38]. There is growing evidence that the anterior insula, whose role as a hub of the salience network is to engage neural networks in response to emotionally important sensory stimuli [39], is affected in ASD [40, 41]. However, the connectivity between the affective anterior insula and sensory posterior insula has not been examined in ASD, nor has the relation between the insula and sensory-affective sequelae that are increasingly considered foundational to the disorder [42, 43].

In this study, we hypothesized that the integrity of structural connectivity in somatosensory and insular regions associated with discriminative and affective touch, respectively, would relate to observed sensory behaviors in a standardized assessment of tactile discrimination and affective responses to touch in young children with ASD. Given the functional posterior-anterior gradient of the insula and the clear differences in affective touch perception [8, 44] and interoception [45–47] in ASD, we specifically examined the structural integrity of intrainsular white matter as a neural substrate for affective response to touch in young children with ASD. As a comparison, we also examined a thalamocortical tract between the ventroposterolateral (VPL) thalamus and primary somatosensory cortex (SI), which is associated with discriminative, rather than affective, touch processing. With a growing body of literature describing differences in discriminative [19] as well as affective response [44] to touch in ASD, we hypothesized that structural connectivity in multiple tracts might differ in ASD [48, 49]. However, given the functional roles of these regions, we predicted that connectivity between the anterior and posterior insula would specifically relate to aberrant *affective* response to touch in individuals with ASD, while connectivity between the VPL thalamus and SI would uniquely relate to aberrant touch *discrimination* in individuals with ASD.

## Methods

### Sample

Twenty-nine children with ASD and 26 typically developing (TD) children between the ages of 5 and 8 years were recruited into the study. This sample is the same as that reported by Pryweller et al. [12]. After excluding participants with poor image quality resulting from excessive motion or scanner/acquisition errors (ASD *n* = 5; TD *n* = 1), the final sample included 23 children with ASD (6.61 years ± 0.89, 2 females) and 24 children with TD (6.58 years ± 1.13, 4 females). Participants in the ASD group were recruited from the university medical center and surrounding community, and a diagnosis of ASD was confirmed with research-reliable administration of the Autism Diagnostic Observation Schedule (ADOS) [50], the algorithm items of the Autism Diagnostic Interview-Revised (ADI-R) [51], and the judgment of a licensed clinical psychologist based on DSM (4th ed.; DSM-IV) [52] criteria. Participants in the TD group were excluded if they had a diagnosed psychiatric or learning disorder or a first-degree relative with ASD. Additionally, TD participants were screened using the Social Communication Questionnaire (SCQ) [53] and the Child Behavior Checklist (CBCL) [54] to rule out risk for ASD and other psychiatric conditions. All participants were screened and excluded for any genetic and neurological problems, previous head injuries, and MRI contraindications.

Participants’ cognitive ability was assessed by trained research assistants using the Kaufman Brief Intelligence Test, Second Edition [55] (*n* = 47; KBIT-2) or WISC-IV [56] (*n* = 1; Weschsler et al. 2003), or estimated (*n* = 1) with the Mullen Scales of Early Learning (MSEL) [57], dependent on the language level of the participant. Although the groups did not differ on sex (*χ* ^2^ (1) = .145, *p* = .70) or chronological age (*t*(43.3) = .085, *p* = .932), Full Scale IQ estimate was significantly higher in the TD group (*t*(28.8) = −4.01, *p* = .0004). See Table 1 for a summary of participant characteristics. Because IQ differed between groups, it was considered as a potential covariate; however, IQ was not significantly correlated with any diffusion variables.

**Table 1:** Sample demographics

	ASD	TD	*p* value
*N* scanned	29	26	–
*N* retained after QA	23	24	–
Mean age in years (SD)	6.61 (.89)	6.58 (1.13)	0.932
FSIQ	98 (19.3)	117 (12.1)	*.0004*
Mean QA rating (SD)	4.21 (0.60)	4.54 (0.64)	0.08
*N* female	2	4	0.703
Mean ADOS severity score (SD)	7.65 (2.06)	–	–

*p* values represent independent sample *t* tests for age, QA, and FSIQ, chi-square test for sex. Significant *p* values are in italics

*SD* standard deviation, *FSIQ* Full Scale IQ, *ADOS* Autism Diagnostic Observation Schedule, *ASD* autism spectrum disorder, *TD* typically developing

### Sensory assessment

Each child was administered the Tactile Defensiveness and Discrimination Test-Revised [12] (Baranek, 2010, unpublished manual). The Tactile Defensiveness and Discrimination Test-Revised (TDDT-R) is a structured observational assessment including internally controlled (active) touch (e.g., using the palm to rub chalk drawings from a square of carpet, digging pennies out of a box of sand) and externally controlled (experimenter-administered, passive) touch (e.g., light touch to the arm, face, or hand with a cotton swab, air puffs administered to the nape of the neck). Affective behavioral responses are scored for (1) tactile defensiveness both to passive, externally controlled/experimenter-administered touch and to active, internally controlled touch (i.e., haptic exploration of materials such as fabric, sand, or putty) and (2) sensory seeking responses (e.g., squealing with delight while enthusiastically playing with sand, repetitively touching fabric), reflecting unusual interest in tactile stimuli. Defensiveness to externally controlled items is scored using a Likert scale from 0–3, with 0 representing no aversive reaction, and 3 representing negative affect that includes crying or retreating from the stimulus. Defensiveness to internally controlled items is a composite of two scores, one assessing approach or avoidance of the stimulus (0–2 scale) and the other a binary score assessing aversive reaction. Total defensiveness is calculated as a composite (sum) variable collapsing across defensiveness scores for both internally and externally controlled stimuli. For internally controlled items, sensory seeking is scored using a binary present/absent score. Discriminative behavioral responses are scored for passive, externally controlled touch (i.e., localization of the body part touched by experimenter) and active, internally controlled touch (i.e., correct haptic identification of shapes or forms handled by the participant).

### Image acquisition and preprocessing

All images were acquired during a single scan session on a 3 Tesla Philips Achieva MRI scanner (Philips Healthcare, Inc., Best, Netherlands). During scanning procedures, participants wore foam earplugs in both ears and Philips headphones to attenuate noise and watched a video of their choice for the duration of the scan. A high-resolution T1-weighted anatomical volume (TR = 9 ms, TE = 4.6 ms, FOV = 256 mm [2], 1 mm isotropic voxels, 170 sagittal slices, 6 min 30 s duration) was collected to provide a template for image registration. Diffusion-weighted data were acquired using a high angular resolution diffusion imaging (HARDI) sequence (2.5 mm [2] isotropic voxels, 50 axial slices, 14 min 34 s duration). Ninety-two diffusion directions (*b* = 1600 s/mm [2]) and one T2-weighted volume (*b* = 0 s/mm [2]) were collected.

Images were visually inspected for common artifacts such as fat shift and ghosting and underwent standard preprocessing and quality assurance procedures that incorporated head motion, artifact propensity, variance, and bias of estimated measures [58]. A QA rating that weighted each of these measures was assigned, with values between 1 and 5. Only scans with ratings equal to or higher than 3 were included in the analysis (as in (12)). The final sample included in the analysis did not significantly differ in this comprehensive QA metric by group (*t*(45) = −1.79, *p* = .08). HARDI data were eddy current-corrected, motion-corrected, and skull-stripped using FMRIB Software Library (FSL) [59, 60]. Fractional anisotropy (FA) maps, eigenvectors, and eigenvalues were created through the DTIFIT module within FSL.

### Probabilistic tractography

**Fig. 1:**
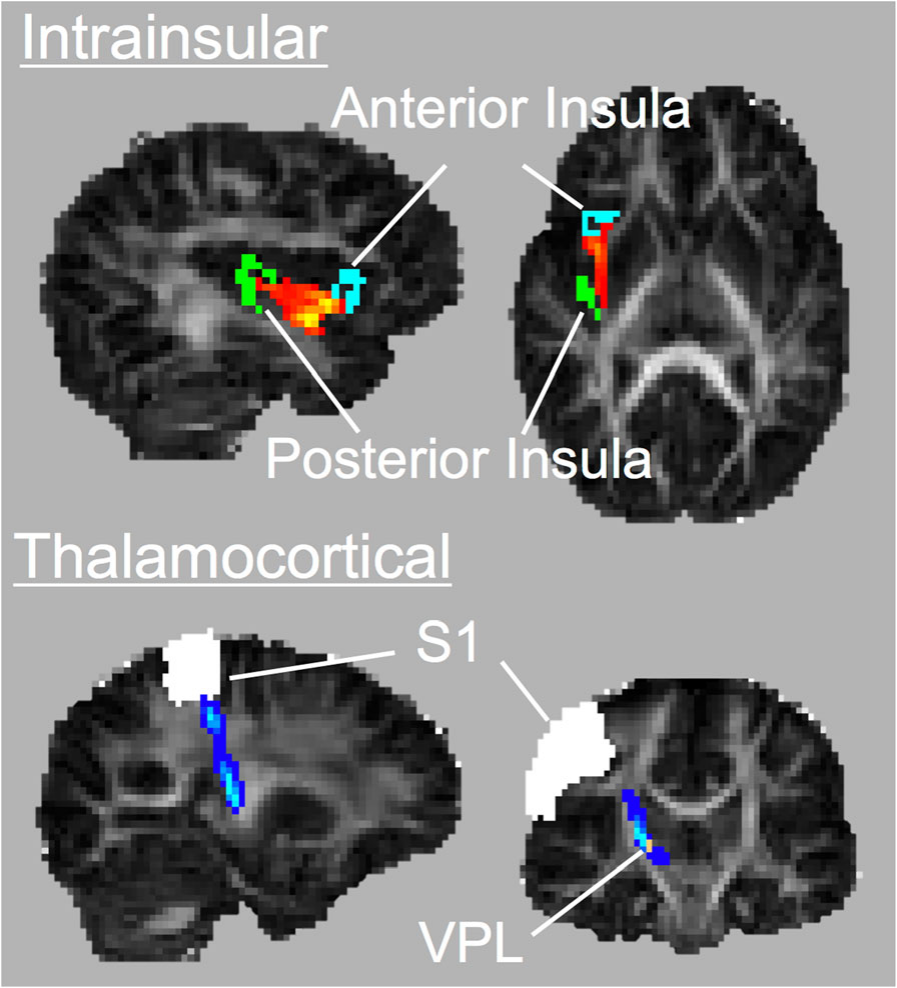
Representative probabilistic distributions of each tract and regions of interest (ROI) used as seed and termination regions for each tract, rendered on fractional anisotropy maps. Intrainsular tract (*top*, *red-orange*) was seeded from the posterior insula (*green*) to the anterior insula (*blue*). The thalamocortical tract (*bottom*, *blue-light blue*) was seeded from the ventral posterolateral nucleus of the thalamus (*VPL*, *yellow*) to primary somatosensory cortex (*SI*, *white*)

Diffusion parameters were estimated at each voxel through the Bayesian Estimation of Diffusion Parameters Obtained using Sampling Techniques (BEDPOSTX) tool in FSL, which uses Markov chain Monte Carlo sampling techniques to estimate diffusion parameters [61]. Within the right hemisphere, two tracts were defined: an intrainsular tract between anterior and posterior insula and a thalamocortical tract between the VPL nucleus of the thalamus and SI (Fig. 1). For the intrainsular tract, anterior and posterior insular seeds (Fig. 1) were traced with a protocol similar to Farb et al. [38] on a pediatric template derived from 324 children ages 4.5–8.5 [62, 63]. The insula was divided into distinct regions based on cytoarchitectonically defined subdivisions among the insular gyri. The anterior seed mapped onto the anterior accessory gyrus, and the posterior seed mapped onto the posterior long gyrus of the insula. For the thalamocortical tract, VPL was defined by the Oxford Thalamic Connectivity Atlas [64], and SI was defined as the post-central gyrus from the Laboratory of Neuroimaging (LONI) probabilistic atlas gray matter tissue map, excluding voxels below a threshold of 15% probability [65]. VPL and SI ROIs were then registered to the pediatric template, and all ROIs subsequently were registered into native space for each subject using a combination of FSL’s linear image registration tool and linear and non-linear Advanced Normalization Tools (ANTs) [66, 67]. Using these seeds and diffusion parameters generated by BEDPOSTX, probabilistic tracking was conducted using FSL’s ProbTrackX (curvature threshold = 0.2; 5000 samples) one-way condition with VPL and posterior insula as seeds and SI and anterior insula as respective waypoints and termination masks. For the larger thalamocortical tract, low-probability voxels were rejected at a threshold of 1%, similar to previous studies [68, 69]. Tracts were visually inspected to confirm that these thresholds reduced noise while producing viable tracts for analysis. Mean fractional anisotropy (FA), mean diffusivity (MD), and volume of each resultant tract were calculated for each thresholded tract. Tract volumes were normalized for each participant by dividing the value by the total brain volume (total white matter, gray matter, and cerebrospinal fluid on skull-stripped T1-weighted anatomical images).

### Statistical analysis

Our primary questions were (1) whether the integrity of white matter in the intrainsular and thalamocortical tracts differs between children with ASD and TD and (2) whether white matter integrity differences in one or both tracts are associated with aberrant affective and discriminative behavioral responses to touch in individuals with ASD, as measured by the TDDT-R. For all diffusion tensor imaging (DTI) variables (FA, MD, and volume in each of the two tracts), we removed outliers (0–2 datapoints per variable) based on values that were extreme (3 times interquartile range). To address question 1, we assessed group differences in DTI variables (FA, MD, volume) for each tract using two sample *t* tests. Although it was not a primary research question, we also assessed group differences in variables derived from the TDDT-R: tactile haptic form perception (discriminative touch), tactile defensiveness (negative affective response to touch) to both internally and externally controlled touch, and tactile seeking (presumed positive affective response to touch). Mann–Whitney tests were used for these comparisons because TDDT-R variables were non-normally distributed (Table 2).

**Table 2:** Median (interquartile range) scores by group on the Tactile Defensiveness and Discrimination Test-Revised (TDDT-R)

Tactile ability/response	TDDT-R	ASD	TD	*p* value
Discrimination	Form perception	12.17 (3.92)	9.71 (4.22)	*0.0125*
Affective response	Defensiveness to externally (experimenter) controlled touch	0.1875(.03)	0.0625 (.13)	*0.0257*
	Defensiveness to touching objects or materials	0.2143 (.25)	0.1429 (.14)	*0.0339*
	Tactile seeking	0.2857 (.14)	0.0000 (.14)	*.0003*

Externally controlled touch is the touch administered by the examiner; internally controlled touch is tactile exploration of objects or materials by the participant. For form perception, higher scores indicate poorer performance (i.e., more time manipulating objects needed to accurately discriminate form). For all other tests, higher scores indicate more observable affective responses to sensory probes. *p* values represent Mann–Whitney *U* tests. Significant *p* values are in italics

*ASD* autism spectrum disorder, *TD* typically developing

To address question 2, we performed nonparametric (Spearman) correlations only between variables (both DTI and behavioral) that had significant differences by group (MD in both tracts and all three TDDT-R variables, see results below). Limiting comparisons to those for which the groups differed significantly allowed us to address the contingency of question 2 on question 1 and to reduce the number of tests performed. To understand how the group interacted with MD in each tract to influence behavior, we conducted a multiple linear regression with the relevant behavioral response score from the TDDT-R as the dependent variable.

To address the possibility of type I error due to multiple comparisons, we conducted permutation testing for significant group comparisons of DTI and TDDT variables as well as TDDT and DTI correlations [70]. A null distribution was calculated for each test statistic across each permutation (*n* = 5000). A *p* value was determined based on the proportion of more extreme test statistics compared to the observed test statistic (*α* = 0.05). The second *p* value reported for the statistically significant findings reported below reflect this corrected value.

## Results

### Tractography

**Fig. 2:**
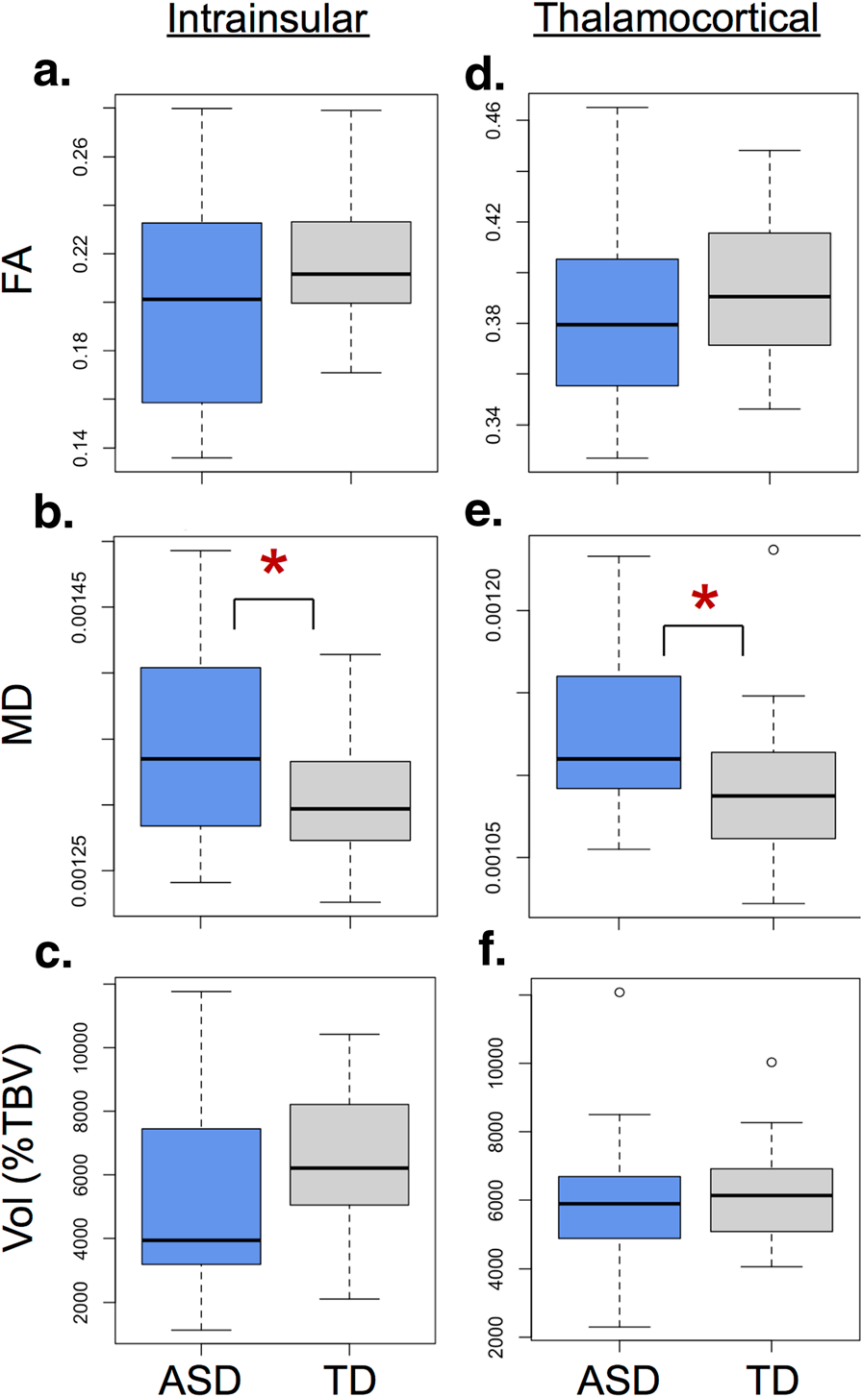
Group differences in tractography. **a**–**c** Boxplots by group of intrainsular tract variables. **a** While FA was lower in individuals with ASD, this was not significantly different compared to that in TD (*p* = .116). **b** MD was significantly higher in the ASD group, compared to that in the TD group (*p = .039). **c** Volume was lower in individuals with ASD, but was not significantly different compared to that in TD (*p* = .084). **d**–**f** Boxplot distributions by group of thalamocortical tract variables. **d** While FA was lower in individuals with ASD, this was not significantly different compared to that in TD (*p* = .183). **e** MD was significantly higher in the ASD group, compared to that in the TD group (*p = .026). **f** There was no significant group difference in volume. *ASD* autism spectrum disorder, *TD* typical development, *FA* fractional anisotropy, *MD* mean diffusivity, *TBV* total brain volume

For both the intrainsular and thalamocortical tracts, MD was significantly higher in the ASD group (intrainsular MD *t* = 2.14, *p* = .039 (permuted *t* distribution (*n* = 5000), *p* = 0.031)); thalamocortical MD *t* = 2.3, *p* = .026 (permuted *t* distribution (*n* = 5000), *p* = 0.024); Fig. 2b, e). Intrainsular volume was marginally lower in the ASD group (*t* = −1.77, *p* = .084), and both intrainsular (*t* = −1.61, *p* = .116) and thalamocortical (*t* = −1.35, *p* = .183) FA showed slight trends to be lower in the ASD group (Fig. 2), consistent with a pattern of reduced tract integrity in ASD. There were no significant group differences or trends in thalamocortical volume.

### Discriminative and affective responses to touch

As measured by the TDDT-R, the ASD group exhibited significantly poorer internally controlled (form) haptic discrimination (*W* = 384.5, *p* = .0215 (permuted *W* distribution (*n* = 5000), *p* = 0.017)) relative to the TD group. In ASD relative to TD, defensiveness to internally controlled tactile stimuli (haptic exploration of materials such as sand or putty) was significantly higher (*W* = 375, *p* = .0339, (permuted *W* distribution (*n* = 5000), *p* = 0.028)), and defensiveness to externally controlled touch (e.g., experimenter touch with a cotton swab) was also higher (*W* = 378.5, *p* = .0257 (permuted *W* distribution (*n* = 5000), *p* = 0.026)). The ASD group exhibited significantly greater tactile seeking behaviors (*W* = 438.5, *p* = .0003 (permuted *W* distribution (*n* = 5000), *p* < 0.001)) relative to the TD group.

### Association between tactile behavioral responses and mean diffusivity

**Fig. 3:**
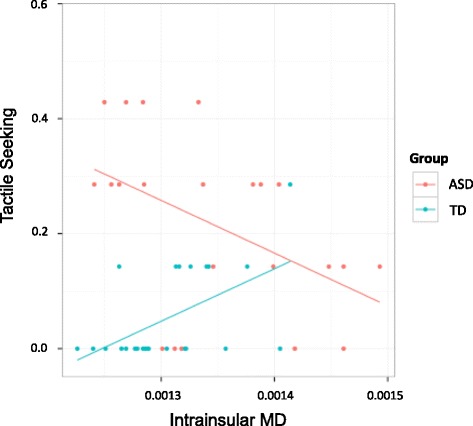
Associations between tactile seeking as measured by the Tactile Defensiveness and Discrimination Test-Revised (TDDT) and intrainsular tractography. Regression lines for ASD (*red*) and TD (*blue*) groups are shown. Significant (Spearman) correlations between intrainsular MD and TDDT-R sensory seeking were observed in the ASD group (*ρ* (20) = −0.50, *p* = 0.0185) and the TD group (*ρ* (22) = 0.49, *p* = 0.0387)

A significant positive association was observed between intrainsular MD and tactile seeking behavior in the TD group (*ρ*(22) = 0.49, *p* = 0.0387 (permuted S distribution (*n* = 5000), *p* = 0.016)), whereas this association was negative in the ASD group (*ρ*(20) = −0.50, *p* = 0.0185 (permuted S distribution (*n* = 5000), *p* = 0.016, Fig. 3)). A Fisher *r*-to-*z* transformation confirmed that these correlation coefficients were significantly different (*z* = 3.47, *p* = .0005). Given the opposite directions of these associations, we then tested for a group*intrainsular MD interaction using a multiple linear regression with tactile seeking as the dependent variable. Full Scale IQ was also included in the model given the group differences in IQ (see Table 1). The model verified significant main effects of group on seeking and showed a main effect of insula MD (*t* (36) = −3.676, *p* = .0008, adjusted total model *r* ^2^ = 0.4647), but not thalamocortical MD or Full Scale IQ (FSIQ), on tactile seeking (see Table 3). There was a significant interaction between group and insula MD (*t* (36) = 3.470, *p* = .0014, adjusted total model *r* ^2^ = 0.4647).

**Table 3:** Linear regression model of tactile seeking (as measured on the Tactile Defensiveness and Discrimination Test-Revised (TDDT-R)) with group, FSIQ, and MD. Total model variance explained, *r* ^2^ = 0.4647

Variable	Estimate	Standard error	*t* value	*p* value
Full Scale IQ	−0.0014	0.0010	−1.299	0.2023
Group	−3.073	0.9852	−3.119	*0.0036*
Insula MD	−2732.22	743.35	−3.676	*0.0008*
Thalamocortical MD	−416.37	1080.47	−0.385	0.7022
Group*Insula MD	1856.07	534.95	3.470	*0.0014*
Group*Thalamocortical MD	438.42	685.19	0.640	0.5263

Significant *p* values are in italics

Neither group exhibited significant associations between tactile discrimination scores and MD in either tract. The TD group showed a negative association between tactile defensiveness and MD in the intrainsular tract (*ρ* (22) = 0.42, *p* = 0.0402 (permuted S distribution (*n* = 5000), *p* = 0.042)); this association was absent in the ASD group.

## Discussion

The goal of this study was to assess the integrity of structural connectivity in somatosensory regions associated with affective and discriminative touch [31] in young children with ASD and to relate it to observed sensory behaviors in a standardized assessment of discriminative and affective responses to touch (Baranek, 2010, unpublished manual). Using the TDDT-R, we observed widespread differences in responses to both affective and discriminative touch in young children with ASD, as has been reported previously [6, 8, 10, 11, 20–22, 71–73]. In the ASD group, we found reduced integrity (increased MD) of the white matter tract connecting the posterior and anterior insula. Based on the roles of these two regions in somatic sensation [32] and affective evaluation [39], respectively, we hypothesize that this tract is likely important for the emotional evaluation of somatic sensory input. The posterior insula receives input from CT afferents [32], a peripheral system that mediates affective touch by responding preferentially to slow, gentle stroking touch on hairy skin. CT afferents are absent from the palmar surface of the hand, suggesting they do not have a role in discriminative touch [74]. Emerging research suggests that individuals with ASD or heightened autistic traits show diminished response to affective touch all along the anterior-posterior axis of the insula [75–77]; the reduced integrity of the intrainsular tract shown by the current study may be a structural basis for this diminished response.

We noted disparate relationships in the two groups between sensory seeking and the integrity of the intrainsular tract, such that higher levels of tactile seeking were associated with *more intact* (lower MD) intrainsular white matter in the ASD group and with *less intact* (higher MD) in the TD group. The frequency of sensory seeking is low in the TD group, and thus, the association with compromised white matter in the insula may reflect aberrant affective responses to touch in typical children and is likely to be of an entirely different etiology than sensory seeking in ASD. The link between sensory seeking and better white matter integrity in the ASD group is intriguing given the debate about the valence of affect in sensory seeking behavior in ASD [9]. While the repetitive nature of the behavior suggests that it is intrinsically reinforcing and there is empirical [78] and autobiographical [79] evidence for association with positive affect, there is also evidence linking the behavior with negative [80] or neutral [9] affect. The association of tactile seeking with greater integrity of a neural pathway that supports positive affective touch supports, but does not prove, the view that sensory seeking reflects positive affect.

It has been hypothesized that sensory seeking behaviors may reflect a compensatory strategy for reduced sensory input [81] or, alternatively, may serve as a coping strategy in response to overwhelming sensory experiences [82]. The possibility that tactile seeking is a compensatory strategy for diminished sensory input may be consistent with association between increased seeking and reduced integrity of the intrainsular tract in the TD group. However, in the ASD group, increased seeking was associated with *greater* integrity of the intrainsular tract. This unexpected profile in ASD—of greater sensory seeking behaviors relating to better integrity of the intrainsular tract—suggests that a more typical level of throughput of sensory information to the salience network may increase the salience of the input disproportionately in ASD, leading to repetitive engagement with a sensory stimulus. Thus, for those individuals with ASD for whom the intrainsular tract is relatively more intact, seeking behaviors could be more effective at increasing sensory input, and thus more self-reinforcing—manifesting as increased intensity or frequency of seeking in a positive feedback loop. This is consistent with the ideas that sensory seeking may overlap with repetitive behaviors more broadly [6, 83] and that many repetitive behaviors appear to be reinforced by affective and reward circuitry [84, 85]. The relatively binary distribution of seeking behavior and its association with a neurobiological variable in our sample suggests its potential for distinguishing meaningful subtypes based on sensory reactivity. Assessing such a subtype in a larger sample might provide additional information about the adaptive function of sensory seeking.

We also observed an association within the TD group between intrainsular tract integrity and overall tactile defensiveness. Somatic input to posterior insula is not limited to the pleasant touch described by Olausson and colleagues; the region responds to interoceptive input [86], unpleasant and painful touch as well [87–89]. Higher defensiveness in the TD group was associated with lower MD, which reflects greater integrity between the posterior and anterior insula. Thus, it is plausible that for TD children, enhanced throughput of unpleasant feelings from the posterior insula to the anterior insula resulted in greater tactile defensiveness. This result should be interpreted cautiously, however, since defensiveness was infrequent in the TD group relative to the ASD group and we did not observe a similar association in the ASD group. The specificity of association between intrainsular connectivity and (presumed) pleasant affective response to touch (seeking) in the ASD group to sensory seeking is of note, suggesting that different neural pathways may contribute to the perception of touch as either pleasant or unpleasant in the altered sensory experience of individuals with autism.

Finally, we did not find any associations between discriminative touch (form perception) and either thalamocortical or intrainsular MD. Indeed, we would not have expected to observe associations between discriminative touch and intrainsular integrity, given that somatosensory projections to the posterior insula are limited to affectively relevant inputs, such as social touch [31] and interoception [37]. While we did predict that group differences in this kind of discriminative touch may be modulated by thalamocortical tract integrity, other neural correlates of form discrimination have also been described [90]. A recent study implicates the serotonergic system in the differentiation of affective versus discriminative touch [91]. Given the importance of serotonin for modulating sensory cortical responses [92, 93], the implication of serotonin in ASD [94, 95], and evidence that the variation in this system specifically impacts somatosensory processing in ASD [10], the relations between autism, serotonin, and affective responses to touch, merit further investigation. Given previous work implicating GABA in altered touch perception [23, 26] in ASD and the modulatory role of serotonin on GABA signaling [96], the interaction of these two neurotransmitter systems in the context of somatosensory perceptual differences in ASD also warrants further study.

Our study had several important strengths, including the use of a standardized observational measure of tactile responsiveness that included quantification of both discrimination and affective response, a high angular resolution DTI sequence that is optimal for tractography, a rigorous QA procedure, and a relatively large and young sample of children with ASD. We utilized a narrow age band of 5–8 years, which is well before pubertal changes in white matter, increases the homogeneity of the sample, and is the earliest developmental look at these tracts in school-age children with ASD. Our study had limitations as well, including the inability to extrapolate our results beyond this narrow age band or to individuals with ASD who could not complete an MRI scan. Ceiling effects or limited variability in some TDDT-R scores for the TD group for some variables may have also hampered our ability to find additional brain–behavior correlations. Finally, while the narrow age band was a strength in some respects, it also limits the ability to extrapolate our findings to the broader population of individuals with ASD. Future research should focus heavily on characterizing behavioral and neural responses to affective touch earlier in development, as the primacy of touch in infancy for a foundation of social reward is well established [97], but not well studied in infants at risk for developing ASD. Understanding how the intersection of perception and affect—both of which have fairly well-characterized neural circuitry—gives rise to the more complex behavioral symptoms of ASD will also depend on strong translational ties between basic and clinical neuroscience. Ultimately, the combination of prospective longitudinal studies of at-risk infants and better cross-talk between basic and clinical researchers is expected to have a strong impact on understanding the pathophysiology of ASD and advancing evidence-based treatment approaches [43].

## Conclusions

This study finds diminished white matter integrity in a group of children with ASD in two tracts conveying somatosensory information. One tract linked the somatosensory VPL nucleus of the thalamus with SI and primarily carries detailed information for discriminative touch. The other tract linked the posterior insula with the anterior insula and would be expected to convey information about the affective nature of somatosensory input. Consistent with previous reports, both discriminative and affective responses to touch were affected in children with ASD. Tactile seeking—defined as enthusiastic and repetitive engagement with a specific sensory stimulus—was associated with the integrity of the intrainsular tract, and the direction of this association differed by group. The findings reported here contribute to our understanding of the neural basis of emotional responses to touch in autism.

## Abbreviations

ADI-R: Autism Diagnostic Interview-Revised
ADOS: Autism Diagnostic Observation Schedule
ANTs: Advanced Normalization Tools
ASD: Autism spectrum disorder
BEDPOSTX: Bayesian Estimation of Diffusion Parameters Obtained using Sampling Techniques
CBCL: Child Behavior Checklist
CT: C-tactile
DSM: Diagnostic and Statistical Manual (American Psychiatry Association)
DTI: Diffusion tensor imaging
FA: Fractional anisotropy
fMRI: Functional magnetic resonance imaging
FSL: FMRIB Software Library
HARDI: High angular resolution diffusion imaging
KBIT: Kaufman Brief Intelligence Test
MD: Mean diffusivity
MRI: Magnetic resonance imaging
MSEL: Mullen Scales of Early Learning
QA: Quality assurance
ROI: Region of interest
SCQ: Social Communication Questionnaire
SI: Primary somatosensory cortex
TBV: Total brain volume
TD: Typically developing
TDDT-R: Tactile Defensiveness and Discrimination Test-Revised
VPL: Ventroposterolateral nucleus of the thalamus
WISC-IV: Weschsler Intelligence Scales for Children. 4th Edition
